# GASTRIC TWIST AFTER SLEEVE GASTRECTOMY: A PROPOSAL FOR ENDOSCOPIC CLASSIFICATION

**DOI:** 10.1590/0102-672020210002e1665

**Published:** 2022-06-24

**Authors:** Luciana T. SIQUEIRA, Fernando SANTA-CRUZ, João Paulo PONTUAL, Maria Amélia R. AQUINO, Luca T. DOMPIERI, Flávio KREIMER, Álvaro A. B. FERRAZ

**Affiliations:** 1 Hospital das Clínicas da Universidade Federal de Pernambuco, General Surgery Service - Recife - PE, Brazil;; 2 Hospital das Clínicas da Universidade Federal de Pernambuco, Postgraduate in Surgery - Recife - PE, Brazil;; 3 Hospital das Clínicas da Universidade Federal de Pernambuco, Gastrintestinal Endoscopy Service- Recife - PE, Brazil;; 4 Universidade Federal de Pernambuco, Medical School - Recife - PE, Brazil

**Keywords:** Bariatric surgery, Endoscopy, Gastrointestinal, Pyloric Stenosis, Cirurgia Bariátrica, Endoscopia Gastrointestinal, Estenose Pilórica

## Abstract

The twisting of the gastric tube is one of the main causes of persistent reflux and food intolerance after sleeve gastrectomy (SG). To date, there is no classification for gastric twist after SG.

**OBJECTIVE:**

This study aimed to propose an endoscopic classification for this condition and outline the clinical profile of these patients with sleeve gastrectomy.

**METHODS:**

Patients in the postoperative period of SG presenting endoscopic findings of gastric twist were included. All patients underwent an esophagogastroduodenoscopy 12 months after SG. The classification proposed consists of three degrees: degree I: mild rotation of the staple line without relevant shrinkage of the gastric lumen; degree II: moderate rotation of the staple line, leading to a focal area of fixed narrowing that requires additional maneuvers for its transposition; and degree III: severe rotation of the staple line leading to stenosis, with increased difficulty for transposition or complete blockage.

**RESULTS:**

Out of 2,723 patients who underwent SG, 45 (1.6%) presented gastric twist. Most patients were female (85%), with mean age of 39±10.4 years. In all, 41 (91.1%) presented degree I, 3 (6.7%) presented degree II, and 1 (2.2%) had degree III. Most patients were asymptomatic (n=26). Vomiting was the most prevalent symptom (15.5%). Statistically significant correlation of twisting degrees was not observed for both the presence of symptoms and the degrees of esophagitis.

**CONCLUSION:**

Gastric twist after SG is rare, with generally mild and asymptomatic presentation. The endoscopic classification was not statistically related to clinical presentation but set the ground for further analysis.

## RESUMO - RACIONAL

A torção do tubo gástrico ou *twist* é uma das principais causas de refluxo persistente e intolerância alimentar após a gastrectomia vertical (GV). Até o momento, não há uma classificação proposta para a torção gástrica após GV.

**OBJETIVO:**

Propor uma classificação endoscópica para essa condição e descrever o perfil clínico desses pacientes.

**MÉTODOS:**

Pacientes no pós-operatório de GV apresentando achados endoscópicos de twist foram incluídos. Os pacientes foram submetidos a uma esofagogastroduodenoscopia 12 meses após a cirurgia. A classificação consiste em três graus. Grau I: rotação leve da linha de grampos, sem redução relevante do lúmen gástrico. Grau II: rotação moderada da linha de grampos, levando a uma área de estreitamento focal que requer manobras adicionais para a progressão do aparelho. Grau III: rotação severa da linha de grampos, levando à estenose, com grande dificuldade de progressão ou obstrução completa.

**RESULTADOS:**

Dos 2.723 pacientes que foram submetidos à GV, 45 (1,6%) apresentaram twist. A maioria dos pacientes era do sex feminino (85%), com idade média de 39±10.4 anos. Quarenta e um (91,1%) apresentaram twist grau I, 3 (6,7%) apresentaram twist grau II e apenas 1 (2,2%) apresentou twist grau III. A maioria dos pacientes eram assintomáticos (n=26). O sintoma mais prevalente foi vômito (15,5%). Não foi observada correlação estatisticamente significante entre o grau de twist e a presença de sintomas ou esofagite.

**CONCLUSÃO:**

Twist após GV é raro e geralmente assintomático. A classificação endoscópica proposta não foi estatisticamente relacionada com apresentação clínica, porém estabelece um referencial para análises futuras.

## INTRODUCTION

Obesity is a chronic, complex, and multifactorial disease that has reached pandemic proportions in the last decades ^3^. Data from the World Health Organization reveals that the prevalence of obesity has tripled since 1975. Today, there are more than 650 million people living with obesity worldwide ^23^. The therapeutic management of obesity involves a multidisciplinary approach, including diet, physical exercise, medications, and surgery. However, bariatric surgery figures as the most effective and lasting treatment option for obesity and its comorbidities, especially for the more severe forms of this condition (body mass index [BMI]=40 kg/m^2^) ^19^.

Currently, sleeve gastrectomy (SG) is the most performed bariatric procedure in the United States, surpassing Roux-en-Y gastric bypass (RYGB) since 2016 ^22^. Despite being fairly similar regarding effectiveness within the short term, some may argue that the global tendency of choosing SG instead of RYGB is controversial as SG presented slightly inferior long-term results in recent randomized controlled trials ^9^ ^,^ ^13^ ^,^ ^20^. In contrast, others advocate that this difference can be compensated by the technical simplicity of SG and the lower risk of postoperative surgical and nonsurgical complications when compared to RYGB ^5^ ^,^ ^12^ ^,^ ^22^.

Notwithstanding its proven safety, SG is related to a non-negligible risk of complications, including food intolerance, gastroesophageal reflux disease, and gastric fistulae ^1^ ^,^ ^8^ ^,^ ^12^ ^,^ ^16^ ^,^ ^22^ ^,^ ^24^. Several mechanisms can contribute to these complications, and the twisting of the remnant stomach, which can occur in 1-10% of cases, appears to play a role on it ^2^ ^,^ ^12^. This morphological alteration creates functional stenosis, blocking food outflow, increasing intragastric pressure, and decreasing the complacency of the remnant stomach ^10^ ^,^ ^24^.

During SG, the greater curvature is completely released from the greater omentum, making the stomach more mobile and prone for twisting ^4^ ^,^ ^25^. Another mechanism for occurrence of twist is sleeve scarring with adhesion formation, leading to a kinking of the gastric tube at the *incisura angularis* ^7^ ^,^ ^17^.

The literature is scarce regarding clinical features and management options for gastric twisting after SG. What we currently have is a small number of series with the most varied end points, hampering any robust analysis on the theme. Considering this scenario, aside from the fact that there are no classifications for gastric twist, our objective was to propose an endoscopic classification for morphological alteration of the gastric tube after SG and present the clinical features and management options for these patients in our center.

## METHODS

### Study design and sample selection

This retrospective study included patients who underwent laparoscopic SG between 2010 and 2019 and presented twisting of the remnant stomach at a late postoperative endoscopic evaluation. Esophagogastroduodenoscopy (EGD) was routinely performed in all patients 12 months after surgery. Those who presented dyspeptic symptoms, persistent vomiting, or hematemesis were submitted to this procedure at the time of presentation, besides the 12 months that followed evaluation.

Age, gender, BMI, comorbidities, the presence of signs and symptoms, gastric twist classification, complications, diameter of the incisura angularis, esophagitis presence, and treatments proposed were collected for study. Data were gathered in an electronic database. The research protocol was approved by the Ethics Committee of our institution under the protocol CAAE 17213819.7.0000.8807.

**Figure 1 -:**
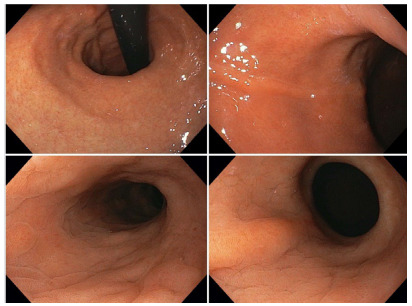
Perfectly symmetrical gastric sleeve, with no signs of twist.

Gastric twist was defined as an axial rotation of the gastric tube. During EGD examination, it can be identified as a clockwise rotation of the staple line, leading to different degrees of shrinkage of the gastric lumen at the level of the incisura angularis. Adequate sleeve is characterized by a straight and symmetrical staple line, without deviations (Figure 1).

**Figure 2 -:**
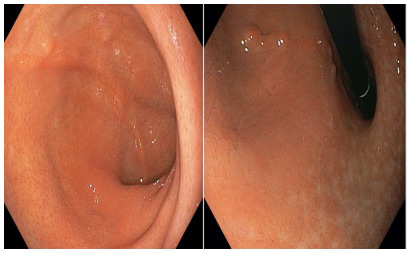
Degree I: mild rotation of the staple line of the remnant stomach without relevant shrinkage of the gastric lumen.

The classification proposed is purely endoscopic and consists of three different degrees of twisting:

Degree I: mild rotation of the staple line of the remnant stomach without relevant shrinkage of the gastric lumen (Figure 2)

**Figure 3 -:**
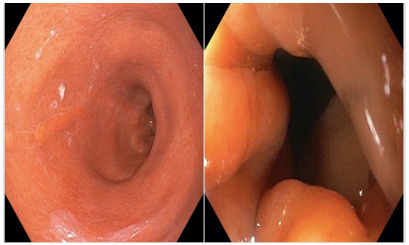
Degree II gastric twist: moderate rotation of the staple line leading to a focal area of fixed narrowing that requires additional endoscopic maneuvers for its transposition.

Degree II: moderate rotation of the staple line, leading to a focal area of fixed narrowing that requires additional endoscopic maneuvers for its transposition (Figure 3)

**Figure 4 -:**
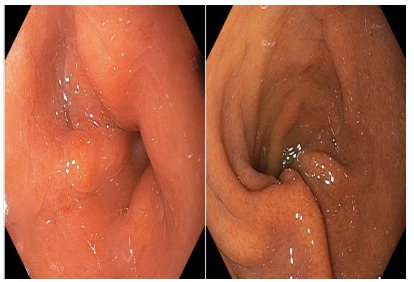
Degree III: severe rotation of the staple line leading to stenosis, with increased difficulty for transposition or complete blockage.

Degree III: severe rotation of the staple line leading to stenosis, with increased difficulty for transposition or complete blockage (Figure 4)

### Surgical technique

We begin by dissecting and removing the fat pad of the gastroesophageal junction. After total release of the greater curvature using ultrasonic energy, we proceed with stapling, initiating in the antrum region, 4 cm from the pylorus, with a 60-mm blue cartridge. The stapler is placed parallel to a 36 Fr *Fouchet* bougie inserted into the stomach. After complete stapling, a transmural continuous suture line is performed with 3-0 *PDS* ^*®*^ along the stapling line. Finally, omentopexy is performed in the distal two-thirds of the gastric tube.

### Endoscopic evaluation

The endoscope used was a 2.8-cm Pentax EG 2990i (Pentax Medical Company, NJ, USA). The main landmark to determine the degrees of twisting was the rotation of the staple line at the level of the incisura angularis. The diameter of the *incisura angularis* was measured through the distance between anterior and posterior gastric walls by using a retrieval forceps of 13.0 mm with maximum insufflation. Esophagitis was graded according to the Los Angeles classification, as follows: (1) mucosal breaks 5 mm without continuity across mucosal folds; (2) mucosal breaks >5 mm without continuity across mucosal folds; (3) continuous mucosal breaks between two mucosal folds involving <75% of the esophageal circumference; and (4) mucosal break(s) involving 75% of the esophageal circumference ^15^.

### Statistical analysis

As part of data analysis, a database was created using Microsoft Excel and exported to STATA/SE version 12.0, in which analysis was performed. Descriptive statistics were used to summarize patient baseline characteristics. Summary data according to the degree of twisting (I-III) are also presented. Quantitative variables are presented as mean, standard deviation, and range; categorical variables are presented as number and percentage. To analyze the association between categorical variables, chi-square test was used. All conclusions were made considering a significance level of 95%.

## RESULTS

The study included 2,723 patients who underwent SG within the study period in our Institution, of which 45 (1.6%) presented gastric twist in the postoperative endoscopic evaluation and were included in the final analysis. The majority of patients were female (85%), with mean age of 39±10.4 years. Mean preoperative and postoperative BMIs were 40.0±3.3 and 27.8±3.3 kg/m^2^, respectively. Hypertension was present at baseline in 40% of the sample, type 2 diabetes (T2D) in 13%, and dyslipidemia in another 26%. All patients were submitted to an EGD before and after (~12 months) surgery. In the preoperative EGD, 37 (82.2%) patients presented normal endoscopic findings, while 8 (17.7%) presented signs of grade A esophagitis. In the postoperative EGD, 71.1% of patients presented normal findings, 20.0% grade A esophagitis, 4.4% grade B esophagitis, and 4.4% grade C esophagitis (Table 1). There were no cases of death in the study period.

**Table 1 -:** Patients’ characteristics.

Sex	Female: 38 (85%) / Male: 7 (15%)
Age (mean ± SD)	39±10.4
BMI before surgery (mean ± SD)	40.0±3.3
BMI after surgery (mean ± SD)	27.8±3.3
Hypertension, n (%)	18 (40)
Type 2 diabetes, n (%)	6 (13.7)
Dyslipidemia, n (%)	12 (26.7)
Preoperative EGD, n (%)	
Normal	37 (82.2)
Grade A	8 (17.7)
Postoperative EGD, n (%)	
Normal	32 (71.1)
Grade A	9 (20.0)
Grade B	2 (4.4)
Grade C	2(4.4)

Notably, 41 (91.1%) patients presented degree I of gastric twist (Figure 2), 3 (6.7%) degree II (Figure 3), and 1 (2.2%) presented degree III (Figure 4). The majority of patients with gastric twist were asymptomatic (n=26). Vomiting was the most prevalent symptom, occurring in seven patients, followed by epigastric pain and dyspeptic symptoms, each referred by four patients. Gastric fistula occurred in only one (2.4%) patient who presented degree II of gastric twist (Table 2). The group presenting degree I gastric twist comprised >90% of the sample.

**Table 2 -:** Clinical presentation according to the degree of twisting.

Clinical presentation	Gastric twist			
	Degree I (%)	Degree II (%)	Degree III (%)	TOTAL
Epigastric pain	4 (9.7)	-	-	4
Weakness	1 (2.4)	-	-	1
Hematemesis	1 (2.4)	-	-	1
Dyspeptic symptoms	4 (9.7)	-	-	4
Refractory dyspepsia	1 (2.4)	-	-	1
Vomiting	5 (12.2)	1 (33.3)	1 (100.0)	7
Fistula	-	1 (33.3)	-	1
No symptoms	25 (61.0)	1 (33.3)	-	26

Table 3 shows the correlation between the presence of symptoms and the degrees of gastric twist and esophagitis. Statistically significant difference was not observed regarding the presence of symptoms according to each degree of twisting or degree of esophagitis.

**Table 3 -:** Presence of symptoms according to each degree of gastric twist and esophagitis.

	Asymptomatic	Symptomatic	Total	p-value
Gastric twist				
Degree I	25	16	41	0.678
Degree II	1	2	3	0.391
Degree III	0	1	1	0.373
Esophagitis				
Degree A	5	4	9	0.892
Degree B	0	2	2	0.208
Degree C	0	2	2	0.208

Table 4 shows the correlation between the degrees of esophagitis and twisting. The majority of patients with evidence of esophagitis in the postoperative EGD presented degree I of gastric twist. However, this result might not be significant as this group comprised the great majority of the sample. Yet, statistically significant difference was not observed between the degrees of esophagitis grades according to each degree of gastric twist.

**Table 4 -:** Correlation between postoperative endoscopic findings (degrees of esophagitis grades and degrees of gastric twist).

Esophagitis	Gastric twist				
	Degree I	Degree II	Degree III	Total	p-value
Degree A	9	-	-	9	0.644
Degree B	1	-	1	2	0.544
Degree C	2	-	-	2	0.744

Correlation between the diameter of the incisura angularis and the degrees of gastric twist is shown in Table 5. Incisura of 10 cm was more prevalent in the group with degree II of gastric twist (p<0.001). Diameters greater than 10 cm did not present statistically significant difference between the groups.

**Table 5 -:** Diameter of the incisura angularis according to each degree of gastric twist.

**Diameter of the *incisura angularis* (mm)**	Gastric twist				
	Degree I	Degree II	Degree III	Total	p-value
10	-	2 (66.7)	-	41	<0.001
11-15	37 (90.2)	-	1 (100.0)	3	0.255
>15	4 (9.8)	1 (33.3)	-	1	0.463

Table 6 describes the therapeutic strategies according to each degree of twisting. The great majority (n=31) of patients did not need any intervention for not presenting symptoms. Eleven patients initiated clinical treatment with proton-pump inhibitor (PPI). One patient in the group of degree III of gastric twist needed endoscopic treatment with balloon dilation. One patient (degree II of gastric twist) needed conversion to RYGB for presenting refractory gastric fistula.

**Table 6 -:** Therapeutic management for each degree of gastric twist.

Treatment	Gastric twist			
	Degree I	Degree II	Degree III	Total
Expectant	31 (75.6)	1 (33.3)	-	32
Clinical treatment*	10 (24.4)	1 (33.3)	-	11
Balloon dilation	-	-	1 (100.0)	1
Conversion to RYGB	-	1 (33.3)	-	1

*Proton-pump inhibitor.

## DISCUSSION

Sleeve gastrectomy is a safe and effective bariatric surgery that is technically simple and has low complication risk. As aforementioned, twisting of the remnant stomach is a relatively rare condition that can create stenosis for the progressive rotation of the staple line, leading to impaired gastric emptying and persistent reflux ^24^. The most common location of twisting is the *incisura angularis*, but it can also occur in the gastroesophageal junction ^7^ ^,^ ^17^.

The incidence of gastric twist following SG is still poorly reported in the literature. Abd Ellatif et al. described 45 cases out of 3,634 patients submitted to SG, showing an incidence of 1.23% of gastric twist ^1^. Out of a total of 860 patients who underwent SG, Hassan et al. found a relatively higher number of gastric twist cases, reporting an incidence of 2.5% ^15^. In our study, we found 45 gastric twist cases out of 2,723 patients who underwent laparoscopic SG, outlining overall incidence of 1.6%.

The time elapsed between the performance of SG and the onset of symptoms is varied, with reports of early and latter presentations, ranging from 25 to 259 days ^1^ ^,^ ^14^. In the present study, we could not evaluate the time elapsed between SG and the onset of symptoms, as the great majority of our gastric twist cases were diagnosed in 1 year following EGD. Only patients with severe clinical presentations (e.g., refractory dyspepsia symptoms, hematemesis, persistent vomiting, and fistula) were submitted to an early EGD.

Nausea, non-bilious vomiting, dysphagia, and regurgitation are reported as some of the most peculiar symptoms related to gastric twist, caused by functional stenosis ^1^ ^,^ ^15^. In our study, the majority of the sample was asymptomatic and statistically significant correlation between symptomatology and the degrees of gastric twist was not observed. The lack of significance may have been caused by the exceedingly small number of patients with gastric twist degree II (n=3) and degree III (n=1), hampering to establish any reliable association. The absence of correlation between the endoscopic classification proposed and the clinical presentation does not weaken this study, as it still provides a standardized and objective tool for further studies on gastric twist. Moreover, lack of statistical significance may be compensated with larger samples, including a higher number of patients with degrees II and III.

Twisting of the gastric tube may also lead to a fearful complication of SG, the gastric fistula. Studies have pointed that kinking of the gastric tube causes upstream pressure, contributing for the onset and persistence of gastric leaks ^11^ ^,^ ^18^. Caiazzo et al., who studied 100 patients with gastric leaks following SG, found that gastric twist was present in 9.0% of their cases, besides being implicated as a predictive factor for conversion to RYGB, given the high rates of therapeutic failure with endoscopic management in these patients ^6^. In our sample, only one case of gastric fistula was observed, which occurred in a patient with degree II twist. This patient was firstly approached for surgical drainage of the abdominal cavity and subsequent insertion of an endoscopic stent inside the gastric tube. As the fistula was shown to be refractory to the conservative management, the surgical team opted for a revisional surgery, converting the SG to an RYGB.

Treatment options for gastric twist include observation/expectant management, balloon dilation, endoscopic stent insertion, seromyotomy, and revisional surgery ^1^ ^,^ ^21^. There is no consensus regarding what would comprise the treatment of choice for gastric twist and the results presented in the literature are highly heterogeneous ^10^ ^,^ ^17^. In our sample, the great majority of patients did not receive any treatment for being asymptomatic. Patients with degree I twist presenting symptoms related to this condition were approached with clinical treatment with PPIs, achieving adequate control of symptoms. As aforementioned, one patient with degree II twist required conversion to RYGB for a refractory gastric fistula. The only patient with degree III twist underwent dilation with an achalasia balloon, achieving early improvement of the clinical complaints (persistent vomiting), with no further complications related to the procedure.

Abhishek et al. proposed an algorithm of treatment for gastric stenosis after SG including in their sample only symptomatic patients and following a sequence of balloon dilation (maximum of four dilations) > endoscopic stents > surgery ^2^. Through application of the patient assessment of upper gastrointestinal symptoms (PAGI-SYM) questionnaire, they found that endoscopic strategies alone succeeded in 88.2% of their sample. Despite having a small sample, their study is of paramount importance for being the first to propose an algorithm to manage gastric stenosis. However, they applied the same intervention sequence (e.g., all patients underwent balloon dilation) for all individuals, regardless of magnitude of twisting. In our study, the majority of patients were asymptomatic and did not need interventions. Only patients with gastric twist of degrees II and III required invasive approaches (conversion to RYGB and balloon dilation, respectively). With our proposed classification, further studies will be able to structure individualized algorithm systems for the management of each degree of gastric twist.

This study has significant limitations: first, related to its retrospective nature and observational intent, requiring further studies to validate the classification proposed and second, the small size of the sample. Gastric twist is a rare entity, so it is difficult to study this complication with a satisfactory number of patients. This could be the reason why the classification proposed did not show correlation with clinical presentation. Furthermore, the study would have benefited from three-dimensional stomach analysis through computed tomography scans in order to complement the information regarding sleeve morphology and external diameter. In contrast, this study has some strengths. It stands as the first proposal of an endoscopic classification of gastric twist after SG. Moreover, all surgeries and EGDs were performed by the same team, contributing to reduce analysis bias related to different technical experiences. This research highlights the importance of studying gastric twist after SG and provides a simple and objective method to standardize the description of this condition in both literature and clinical practice.

## CONCLUSION

Despite not presenting correlation with symptoms presentation, the endoscopic classification proposed for gastric twist provides a standardized description of this condition, facilitating the interpretation of data in the literature from now on and enabling future considerations regarding the optimal management options and successful decision-making for each degree of twisting. Furthermore, it was observed that gastric twist after SG is a relatively rare condition, with generally mild (degree I) and asymptomatic presentation. However, with the progressive rotation of the staple line, a stenosis might occur (degree III). Further evaluation of this classification system is still needed.
